# Teaching critical appraisal to large classes of undergraduate medical students using team-based learning versus group discussions: a randomized controlled trial

**DOI:** 10.1186/s12909-022-03145-9

**Published:** 2022-02-04

**Authors:** Dayane Daou, Marlene Chakhtoura, Ahmed El-Yazbi, Deborah Mukherji, Eman Sbaity, Marwan M. Refaat, Mona Nabulsi

**Affiliations:** 1 Department of Anesthesiology, American University of Beirut, Beirut, Lebanon; 2 Department of Internal Medicine, American University of Beirut, Beirut, Lebanon; 3 Department of Pharmacology, American University of Beirut, Beirut, Lebanon; 4 Department of Surgery, American University of Beirut, Beirut, Lebanon; 5 Department of Pediatrics and Adolescent Medicine, American University of Beirut, Beirut, Lebanon

**Keywords:** Critical appraisal, Evidence-based medicine, Preclinical medical education, Small group discussions, Team-based learning

## Abstract

**Background:**

Evidence on the effectiveness of team-based learning in teaching critical appraisal to large classes of preclinical medical students is scarce. This study investigated whether team-based learning is effective in teaching critical appraisal to large classes of preclinical medical students.

**Methods:**

Between April 2018 and May 2019, 107 first-year medical students were randomly allocated to receive instruction in critical appraisal using team-based learning or traditional group discussions as teaching methods. The primary outcome was students’ performance on the Berlin Questionnaire administered at the end of second year.

**Results:**

Students’ mean (SD) age was 22.0 (0.7) years. Baseline characteristics of the two groups were similar (all *p* values > 0.05). The mean (SD) Berlin scores of both groups were 80.4 (11.6) and 80.1 (12.1) for team-based learning and group discussions, respectively. Multivariate stepwise linear regression analysis revealed that the student’s academic achievement in medical school was the sole predictor of performance on the Berlin Questionnaire (*ß* = 1.079, *p* < 0.001), adjusting for gender, Medical College Admission Test score, student’s self-reported preferred teaching method, rank upon admission to medical school, score on the Epidemiology and Biostatistics course, and teaching method (team-based learning versus group discussions).

**Conclusions:**

Team-based learning and group discussions were equally effective instructional strategies to teach critical appraisal to large classes of undergraduate medical students. Replication of our findings is needed in other educational settings.

**Trial Registration:**

Current Controlled Trials ISRCTN15430424, retrospectively registered on December 30, 2021.

## Background

The Sicily statement on evidence-based practice [1] stated that evidence-based practice “requires that decisions about health care are based on the best available, current, valid and relevant evidence.” Evidence-based practice promotes patient-centered care and application of best practices and is associated with improved physicians’ performance and patients’ outcomes [2]. Hence early training of undergraduate medical students in the skills of evidence-based medicine (EBM) is expected to facilitate their development into future evidence-based practitioners.

The Faculty of Medicine at the American University of Beirut (AUB) recently implemented a new curriculum, the Impact curriculum [3], which includes EBM as one of its competencies. In this curriculum, the teaching of EBM principles starts in first year medical school and continues vertically until graduation at the end of the fourth year. In the first pre-clinical year, students are taught the principles of Epidemiology and Biostatistics in the Fundamentals of Medical Research (FMR) course, at the end of which they start the EBM course. Following an introductory session about the philosophy of EBM and why it is needed, students get instructed on how to phrase clinical questions from clinical scenarios using the PICO format. Critical appraisal (CA) is then taught in each organ system module during first and second pre-clinical years. In third and fourth clinical years students get to apply the whole process of evidence-based practice by conducting “EBM rounds” in Internal Medicine, Pediatrics, and Surgery clerkships on weekly basis. During the rounds, each student is expected to present one of his/her patients, generate a focused clinical question, search electronic databases for the evidence on this question, appraise the evidence, and discuss the application to the patient in terms of benefits, harms, cost, and patient preferences and values.

Undergraduate pre-clinical classes are relatively large (100 to 120 students per class), with seven instructors participating in CA training. Because of the variable instructor availability to conduct group discussions (GD) during any academic year, the class is divided into groups of 14 to 25 students when four or more instructors are available for a particular session. However, when instructors are less than four, CA is taught using Team-Based Learning (TBL) as an instructional method, with one instructor conducting the TBL session for the whole class. Our students are familiar with the TBL approach as they do several such sessions in their first year. TBL is an application-oriented teaching method that incorporates multiple small groups into a setting of a large group, thus combining both small- and large-group learning. TBL requires only one instructor to facilitate learning of the smaller groups simultaneously, thus efficiently utilizing human resources. It has therefore gained popularity in medical education as it can be applied to large groups of up to 100 students, as opposed to smaller group discussions that require the availability of several instructors [4, 5]. Moreover, TBL helps students achieve course objectives while learning how to function in teams [4, 6, 7]. However, TBL as an EBM teaching method was described in only few studies [8–10], reporting high level of students’ engagement and interaction in class, as well as fostering individual accountability, and promoting teamwork behaviors consistent with effective EBM practice [10].

There are different educational strategies to teach EBM in the undergraduate medical curriculum reported in the literature. These include didactic lectures, workshops, small group discussions, online courses, and blended techniques [11–13]. However, no single instructional method appears to be superior in teaching EBM skills, as reported in several systematic reviews that compared the different instructional methods [12, 14, 15]. Well-conducted studies with rigorous designs are thus needed to assess the effectiveness of the different methods for teaching EBM to undergraduate medical students.

To our knowledge, there are no studies with robust designs such as randomized controlled trials (RCTs) that compared the effectiveness of TBL versus GD as CA instructional methods. We therefore conducted this educational quality improvement project (QI), designed as an RCT, to compare the effectiveness of TBL versus GD in teaching CA to undergraduate medical students. The findings from this QI will guide us how best to teach CA to large classes of preclinical medical students, given the limited number of teaching faculty available for such classes.

## Methods

### Design

We conducted this randomized open-label parallel group trial during the academic years 2018-2019 (April 2018 to May 2019) as an educational quality improvement project at the Faculty of Medicine of the American University of Beirut, Lebanon. This project was labelled as QI because the limited numbers of instructors was identified as an issue that may affect the quality of teaching critical appraisal, thus compromising achievement of the course learning objectives. There was a need therefore for an educational intervention such as TBL that can adapt to this challenge without affecting the quality of the teaching. If TBL would prove to be as, or more effective than GD, then it would be the instructional format of choice for future classes when the number of needed instructors is not met. We elected to compare the two teaching formats (TBL vs. GD) head-to-head using an RCT design, instead of the typical pre-post design of QI because of the robustness of the RCT design that would minimize the risk of selection bias. A pre-post design would be confounded by the fact that the students in the pre-intervention phase would be different from the post-intervention phase.

 The Curriculum Committee of the Faculty of Medicine at the American University of Beirut approved this quality improvement project. Students were informed of the aims and procedures of this QI at the beginning of the FMR course. The Institutional Review Board exempted this QI from review. Written informed consent from students for use of their data in this report was obtained.

An independent statistician allocated first year medical students into either TBL or GD groups, using a computer-generated permuted block randomization of variable block sizes. We concealed allocation from students and instructors until the first day of the course. Instructors rotated on groups during the different sessions according to a computer-generated sequence to assure similar student experiences. This QI is reported in accordance with the Revised Standards for Quality Improvement Reporting Excellence (SQUIRE 2.0) [16], and in accordance with the CONSORT 2010 statement guidelines for reporting parallel group randomized trials [17].

### Participants

All first-year medical students in 2018 (N=108 students). The team of instructors consisted of full-time faculty members from the departments of anesthesiology, internal medicine, pediatrics, pharmacology, and surgery who were trained in TBL and in EBM, and who constituted the EBM team at AUB.

### Interventions

We divided students during their EBM sessions in first- and second-year medical school into TBL or GD groups (1:1 ratio) according to the random sequence generated by the computer. In each organ system module, one CA session was designed to address a topic relevant to that module. Table 1 details the list of topics discussed in each module. One week prior to each session, the session’s learning objectives, reading material and student instructions were disseminated to all students. The reading material included references to the paper to be discussed in the EBM session, and the relevant CA chapter from the handbook Users’ Guides to the Medical Literature [23]. Students were instructed to read the CA chapter and try appraising the paper prior to the session. They were also advised to review from their FMR course concepts that are relevant to the content of the planned session as outlined in the session’s learning objectives. Students were informed that they will be administered a 10-minute, multiple choice quiz at the beginning of the session to test their understanding of these concepts.

**Table 1:** List of critical appraisal sessions

Critical Appraisal Topic	Module	Topic
Therapy	Blood	Treatment of iron overload in thalassemia [18]
Systematic review	Gastro-intestinal	Helicobacter eradication and gastric neoplasia [19]
Diagnosis	Respiratory	Diagnosis of pneumonia by ultrasonography [20]
Prognosis	Cardiovascular	Association of body weight and lifestyle factors with mortality [21]
Harm	Psychiatry	Birth weight and risk of Autism [22]

During the session, the TBL group conducted a typical TBL session starting with a 10-minute individual readiness assurance test (IRAT) to assess each student’s basic understanding of the concepts to be further discussed during the session. The TBL group was then divided into smaller subgroups of 6-8 students and were administered the same 10-minute test which they would answer as a group after reaching consensus using scratch-off cards (group readiness assurance test-GRAT). Following the GRAT, the students received immediate feedback on controversial questions through an active discussion between the groups and the instructor. Once all student queries were addressed, the students in each subgroup worked together on an application exercise consisting of the CA of the provided paper. The time allotted for the application exercise was 60 min, after which the instructor facilitated a discussion of CA elements among groups, clarifying controversial issues.

**Fig. 1:**
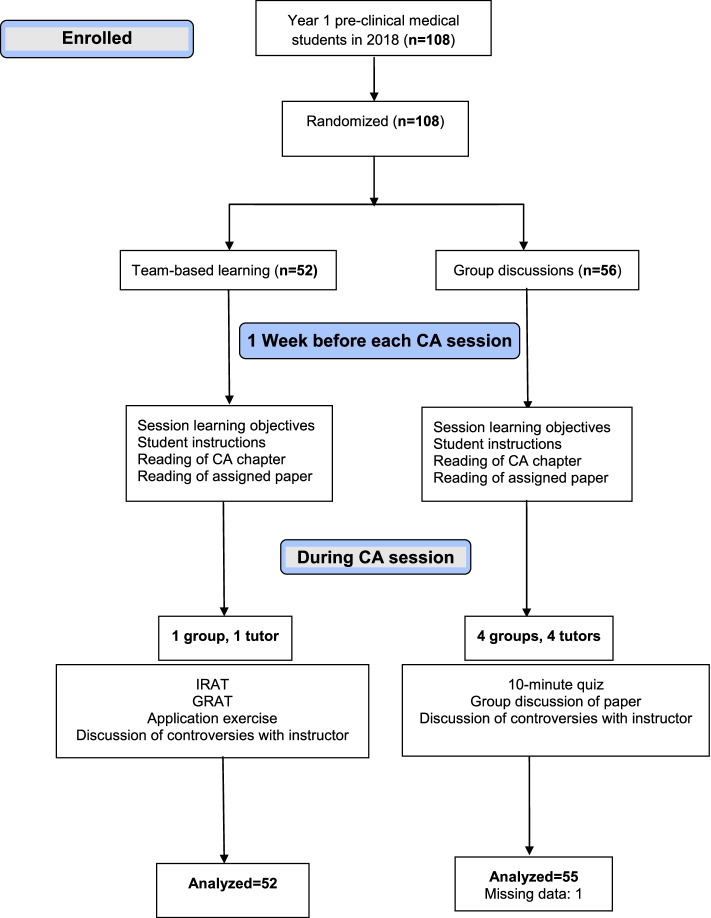
CONSORT flow diagram

In contrast, the GD group was further divided into smaller subgroups of 8-13 students with each subgroup assigned to one instructor. The number of GD subgroups varied from one module to another depending on the availability of the EBM instructors which ranged between four and seven instructors (median of 4 instructors). Each subgroup started the session with the same 10-minute test administered to the TBL group. However, there was no GRAT test later or team work on an application exercise. Instead, the GD subgroup discussed the same paper assigned for the module as a small group discussion, with the EBM instructor facilitating the discussion and interfering only when the subgroup needed redirection of the ongoing discussion, or further clarification of a controversial issue that the students were unable to resolve. The EBM instructors rotated on TBL and GD subgroups during the academic year to assure similar exposure of students to the different instructors. The assignment of the instructor to the TBL group or GD subgroup was done according to a computer-generated random sequence to avoid selection bias. The flow of students through the trial is summarized in Fig. 1.

It is noteworthy to mention that in preclinical years, there is no formal EBM instruction or integration of EBM concepts in parts of the curriculum other than this course.

### Outcomes

Our primary outcome was students’ knowledge in EBM as measured by their performance on the mandatory final exam administered at the end of the second year of medical school. The students were administered the Berlin Questionnaire-Set B as their EBM final exam. This instrument is a validated EBM questionnaire that was developed to assess knowledge and skills in EBM following a short course in EBM. It has two versions (Set A and Set B) with similar content. Each set has 15 questions designed to measure deep learning rather than superficial learning as they are based on clinical scenarios. The Cronbach’s alpha of set B is 0.82 [24].

### Data

The following student variables were used as data for this QI: age, gender, Medical College Admission Test (MCAT) score, rank upon admission to medical school (in tertiles), grade on the FMR course, pooled grade average of all courses at the end of first year excluding FMR, students’ self-reported preferred teaching method (TBL vs. GD) assessed by a survey at the beginning of the FMR course (response rate 100%), and students’ scores on the Berlin Questionnaire (Total score = 100 points).

### Statistical analysis

The class size was 108 students which assured 80% power, with 5% alpha level to detect a difference of 0.55 standard deviations in the mean scores of both groups on the Berlin Questionnaire.

We summarized continuous data as means and standard deviations, and categorical data as counts and percentages. The mean score of the two groups on the Berlin Questionnaire was compared using Student’s independent *t* test. We further investigated the relationship between the Berlin Questionnaire score as outcome and the teaching format (TBL vs. GD) as predictor (forced variable) in a stepwise multivariate linear regression model, adjusting for gender, MCAT score, rank upon admission to medical school, student’s self-reported preferred teaching method, pooled grade average of all courses excluding FMR, and grade on the FMR course as covariates. Data entry, management, and intention to treat analysis were done using IBM SPSS version 24. A *p* value of <5% indicates statistical significance.

## Results

Of 108 students, 52 were randomized to TBL and 56 to GD groups (Fig. 1). This report includes the results of 107 students. One student from the GD group did not submit his written informed consent so his data were excluded from this analysis. The mean (SD) age of the students was 22 (0.7) years, and 57 (53.3%) were females. The preferred method of instruction as reported by the students at baseline was small group discussions for 64 (59.8%), TBL for 34 (31.8%), and didactic lecturing for 9 (8.4%) students. The mean (SD) scores were 509.4 (5.0) points on MCAT, 87.7% (6.3%) on FMR, and 80.2% (11.8%) on the Berlin Questionnaire. In bivariate analysis, no significant differences between GD and TBL groups were found in the baseline characteristics (Table 2), or in the mean scores on the Berlin Questionnaire (TBL = 80.4 ± 11.6, GD = 80.1 ± 12.1; *p* = 0.92).

**Table 2:** Students’ Characteristics and scores on Berlin Questionnaire (N=107)

Student Characteristics	TBL *n*=52	GD *n*=55
**Categorical variables**		
Female gender *n* (%)	24 (42.1)	33 (57.9)
Rank on admission *n* (%) 1st tertile 2nd tertile 3rd tertile	17 (32.7) 19 (36.5) 16 (30.8)	22 (40.0) 19 (34.5) 14 (25.5)
Preferred instruction method *n* (%) GD TBL Lecturing	30 (57.7) 17 (32.7) 5 (9.6)	34 (61.8) 17 (30.9) 4 (7.3)
**Continuous variables**		
Score on MCAT *M (SD)*	509.4 (4.8)	509.5 (5.3)
Score on FMR *M (SD)*	87.4 (6.9)	88.0 (5.7)
Score on courses excluding FMR *M (SD)*	84.3 (5.1)	83.8 (5.1)
Score on Berlin Questionnaire *M (SD)*	80.4 (11.6)	80.1 (12.1)

*Note*. TBL=Team-based learning; GD=group discussions; MCAT=Medical College Admission Test; FMR=Fundamentals of Medical Research course; SD=standard deviation; M=mean

The linear regression model (Table 3) revealed that the method of teaching CA was not a statistically significant predictor of the student’s score on the Berlin Questionnaire (*ß*= 1.079, *p* = 0.90). There was no multicollinearity between the covariates in the model. Interestingly, the only significant predictor in this model was the pooled grade average of all courses at the end of first year, excluding FMR (*ß=* 1.079, *p* < 0.001), after adjusting for gender, student’s preferred instruction method, MCAT score, rank on admission to medical school, FMR score, and group allocation (TBL vs. GD as a forced variable).

**Table 3:** Predictors of Performance on the Berlin Questionnaire in Multivariate Linear Regression Analysis (N=107)

Predictor	*ß*	*95% CI*	*p*
Group allocation	0.27	-3.79 to 4.33	0.900
Score on all courses excluding FMR	1.08	0.68 to 1.48	<0.001

*Note.* FMR=Fundamentals of Medical Research; Variables entered into the model: gender, student’s preferred instruction method, score on all courses excluding FMR, score on Medical College Admission Test, rank on admission to medical school, score on Fundamentals of Medical Research course (FMR), group allocation (team-based learning vs. group discussions) as a forced variable

## Discussion

Teaching CA to pre-clinical medical students is challenging because of their limited clinical skills and medical knowledge. Different teaching interventions have been used to train undergraduate medical students in EBM principles such as standalone workshops or yearlong courses, with variable teaching and learning methods like didactic lecturing, interactive learning, or online learning [12]. A Best Evidence Medical Education (BEME) systematic review [25] that investigated the impact of the different EBM teaching strategies on undergraduate medical students’ knowledge, attitudes, skills, and behaviors found the literature to be of low-quality evidence, with use of non-validated instruments, and lack of robust study designs. Of the different teaching strategies, only e-learning showed potential to improve students’ knowledge, attitudes, or skills. None of the studies in the BEME review included TBL as an EBM teaching strategy.

It is interesting to note that our students’ scores on the Berlin Questionnaire were high, approaching the scores of EBM experts that participated in the validation study of the Berlin Questionnaire (mean of 0.82, SD = 0.29) [24]. This finding is explained by the fact that our students are prepared to learn CA since the course of epidemiology and biostatistics (FMR) is taught in first year of medical school before EBM. Also, the FMR course equips students with the necessary skills to conduct a small research project in teams, as a curricular requirement during the second year.

In this study, we found TBL to be as effective a method for teaching CA to pre-clinical medical students as traditional group discussions. TBL has been shown to be more effective than didactic lectures in improving medical students’ theoretical examination scores, learning attitude, and learning skill [26]. Moreover, it improves students’ end of course grades, classroom engagement, and test performance, with students reporting deeper understanding of the content and better preparation for assessment and course performance [27]. In a study from Singapore, TBL was the students’ preferred method of learning when used for teaching EBM principles to undergraduate medical students, promoting a higher level of students’ engagement in discussion and group interaction than conventional tutorial [9]. A study from Baylor College of Medicine [8] reported that TBL enhanced student accountability and teamwork when used to teach an EBM course to undergraduate medical students. These studies had non-randomized designs, which makes this report the first randomized trial to compare the effectiveness of TBL and GD, head-to-head, as CA teaching strategies in undergraduate medical education.

We found the pooled grade average of all courses (excluding FMR) at the end of first year to be the sole predictor of the students’ scores on the Berlin Questionnaire. This finding implies that beyond the baseline training in CA provided by TBL and GD, the competence in CA is only influenced by the student’s academic achievement in medical school and his/her perseverance, rather than by the teaching strategy used in class. It may be argued that since both TBL and GD groups conducted smaller group discussions to analyze and appraise the paper at hand (as in problem-based learning), the effect of the teaching strategy on the long-term retention of the student as assessed by the Berlin Questionnaire were attenuated. Hence the student’s performance was mostly influenced by his/her academic abilities, which is consistent with evidence indicating that academic or cognitive ability is a moderate to large predictor of success in undergraduate medical training [28, 29]. The fact that GD as a method of instruction was favored by 60% of our students is not surprising, as typically there are no IRATs or GRATs during GD that would incentivize them to prepare well before class. During the discussion, they can always turn to the instructor for explanations, whereas in TBL students explain to each other when working in their small groups on the application exercise. This is when teamwork and peer-to-peer teaching happens. They would resort to the instructor only when faced with controversies or disagreements. In a study that assessed the long-term impact of TBL on our preclinical students of the Impact curriculum, Zgheib, et al. [7] showed that students had long-term and sustained improvement in self-learning and problem-solving, as well as improved team dynamics, professionalism, and personal development.

Our study has strengths and limitations. The main strength of this trial is that the students had similar baseline characteristics which helped minimize the effect of any potential confounder on students’ performance on the Berlin Questionnaire. Moreover, the class size provided enough power to detect small differences in the scores of the two groups, and the fact that the course was a curricular requirement helped avoid attrition bias. The random rotation of the instructors on the different groups allowed exposure of students to facilitators of different backgrounds which may enhance the generalizability of our findings to other settings. Finally, our study assessed long-term retention and application of concepts at the end of second year of medical school, rather than short-term knowledge acquisition at the end of a course or workshop like most of the literature. The main limitation of our trial is the lack of blinding of students and instructors which was unavoidable because of the nature of the teaching strategies. However, the use of a validated questionnaire to measure students’ knowledge of EBM concepts reduced the risks of performance and detection biases.

## Conclusions

TBL may be a useful strategy to teach CA to large classes of pre-clinical medical students instead of traditional group discussions. TBL is especially useful when the number of EBM instructors is limited such as in our institution. Prior knowledge of epidemiology and biostatistics concepts is however necessary for students to achieve good CA skills.

## Data Availability

The datasets used and/or analysed during the current study are available from the corresponding author on reasonable request.

## Abbreviations

EBM: Evidence-based medicine
AUB: American University of Beirut
FMR: Fundamentals of Medical Research
CA: critical appraisal
GD: group discussions
TBL: team-based learning
RCT: randomized controlled trial
QI: quality improvement
SQUIRE: Standards for Quality Improvement Reporting Excellence
CONSORT: CONsolidated Standards of Reporting Trials
IRAT: individual readiness assurance test
GRAT: group readiness assurance test
MCAT: Medical College Admission Test
M: mean
SD: standard deviation
BEME: Best Evidence Medical Education
